# Effects of non-invasive respiratory support on sleep in preterm infants evaluated by actigraphy

**DOI:** 10.5935/1984-0063.20200035

**Published:** 2021

**Authors:** Fernanda Schmidt, Felipe Kalil Neto, Graciane Radaelli, Magda Lahorgue Nunes

**Affiliations:** 1 Universidade Luterana do Brasil, Department of Neonatology - Canoas - RS - Brazil.; 2 Hospital São Lucas da Pontifícia Universidade Católica do Rio Grande do Sul, Department of Neurology - Porto Alegre - RS - Brazil.; 3 Brain Institute of Rio Grande do Sul - Porto Alegre - RS - Brazil.; 4 Escola de Medicina da Pontificia Universidade Catolica do Rio Grande do Sul - Porto Alegre - RS - Brazil.

**Keywords:** Sleep, Actigraphy, Infant, Premature

## Abstract

**Objective:**

Few studies have evaluated sleep in preterm infants under non-invasive ventilatory support in neonatal intensive care units (NICU). The main objective of this study was to evaluate the inﬂuence of continuous positive airway pressure (CPAP) in the sleep of premature babies.

**Material and Methods:**

Crossover study in a NICU. We selected preterm infants with gestational age between 28 and 37 weeks using nasal CPAP. Eighteen preterm were included. Patients were monitored with actigraphy and with the Neonatal Behavioral Assessment Scale (NBAS).

**Results:**

Results showed a reduction in sleep effciency, total sleep time and total sleep period during the CPAP period when compared to the non-CPAP. NBAS demonstrated signiﬁcantly greater time of deep sleep and light sleep in the period without CPAP.

**Conclusion:**

Our data suggests that the use of CPAP, during the ﬁrst week of life, in preterm neonates, is associated with transitory alterations of sleep organization.

## INTRODUCTION

Sleep architecture in premature infants is a complex phenomenon, since the maturation of their brain structures and functions are not always linear^1,2^.

Prematurity may have a major impact on the structure of sleep, including adverse intrauterine exposures and/or stressors in the neonatal period. This impact is explained by the maturation of the suprachiasmatic nucleus occurring in an unfavorable environment, including stressors such as hypoxia, malnutrition and constant luminosity in neonatal intensive care units (NICUs)^3^.

Other studies also demonstrate that several stimuli can cause alterations in sleep architecture, such as handling by the assistant team, changes in body position and use of mechanical ventilation^4,5^.

Additionally, there is evidence that sleep deprivation during brain development results in a reduction of neuronal plasticity, altering learning processes and with long-term effects on behavior and in some brain functions^6-8^.

Improved respiratory support is one of the major factors responsible for the decreased mortality in NICUs, since the beginning of the twentieth century, when the use of continuous positive airway pressure (CPAP) started to be routinely used to stabilize the respiratory disease in preterm infants^9-11^. Currently, randomized controlled trials suggest that the early use of nasal CPAP in very low birth weight infants is associated with a reduction in the need for invasive support^9^.

Knowing the numerous interferences that a premature infant is exposed in the NICU, it is impressive how just a few studies evaluated sleep of premature infants in the use of non-invasive respiratory support^1^. Thus, the objective of this study was to evaluate subject and objective sleep in preterm newborns undergoing continuous positive airway pressure using nasal cannula.

## MATERIAL AND METHODS

Crossover study performed at the NICU of ULBRA (*Universidade Luterana do Brasil*) University Hospital. The study was approved by the Ethics Committee of the Pontifícia Universidade Católica do Rio Grande do Sul (PUCRS) and of ULBRA (number: 66935717.9.1001.5336). A neonatologist calculated gestational age with the New Ballard Method, which includes neuromuscular and physical maturity^12^. All parents/guardians signed the informed consent form. Clinical characteristics of the sample are summarized in Table 1.

**Table 1:** Clinical Characteristics of the sample.

Variables	n=18
Gestational age (weeks) - mean ± SD	31,7 ± 2,1
Days of life with CPAP - median (P25-P75)	2 (1-3)
Days of life without CPAP - median(P25-P75)	5 (4-6)
Sex - n (%)	
Female	8 (44,4)
Male	10 (55,6)
Weight (g) - mean ± SD	1708 ± 416
Delivery - n (%)	
Vaginal	9 (50,0)
Cesarean	9 (50,0)
Premature labor - n (%)	14 (77,8)
Use of corticoid - n (%)	16 (88,9)
Use of surfactant - n (%)	10 (55,6)
Oxygen (FiO2) - mean ± DP	0.319 ± 0.057
Apgar 1 - median (P25-P75)	8 (7-8)
Apgar 5 - median (P25-P75)	8 (8-9)
PEEP ( cmH2O) - mean ± SD	5,33 ± 0,49

SD = Standard deviation; PEEP = Positive end-expiratory pressure.

The actigraphy monitoring occurred within the first week of life in all patients, the first evaluation (with CPAP) occurred in the 2nd day of life (median) and the second (without nasal CPAP) in the 5th (median) day of life.

### Inclusion/exclusion and sample criteria

Inclusion criteria were premature infants with less than 37 weeks and with birth weight over 800g, using oral or intravenous caffeine and undergoing continuous positive airway pressure using nasal cannula. Exclusion criteria were presence of suspected seizures, continuous use of sedative drugs, cerebral malformations, chromosomal disorders, cerebral hemorrhage. Eighteen preterm infants were included in the sample criteria (8 female, 10 male) with a gestational age between 29-36 weeks (31.7 ± 2.1) and birth weight from 840g to 2,340g (1708 ± 416); nine were delivered by a cesarean section.

### Procedure and evaluations

Patients were evaluated in two moments, initially using nasal CPAP with the Nellcor Puritan Bennet machine respirator, using nasal cannula and later, the same patients, were evaluated without nasal CPAP. All analysis were performed during the first week of life.

At first, the actigraph was placed randomly in the right or in the left leg (the side oscillated according to the oximeter device) for 48 hours. The actigraph was attached with a cotton bandage. Within this period, the Neonatal Behavioral Assessment Scale (NBAS) as proposed by Brazelton and Nugent^13^ was performed during four intercalated 30-minute episodes, totaling 2 hours of observation. The same researcher carried out this evaluation. Sleep diary was used to exclude staff handling and feeding periods.

The *Micro Motionlogger from Ambulatory Monitoring Inc.* was held for 48 hours in the leg, between the knee and ankle under a cotton bed. The observed actigraph patterns were total sleep period (TSP) - defined as the number of minutes from onset of sleep to awakening; total sleep time (TST) - characterized as the number of minutes of the sleep time excluding the awakenings; sleep efficiency - the ratio between TST and TSP, expressed as a percentage^14-16^.

Immediately after CPAP removal, the same evaluation was repeated for a further 48 hours.

Sleep rating by the NBAS is divided in six stages such as: deep sleep (closed eyes, regular breathing, no spontaneous activity, no eye movement); light sleep (closed eyes, irregular breathing, low activity level, eye movements may be present, suctioning or occasional movements); sleepy (open eyes, heavy eyelids, variable activity level, responsive to sensory response with slow response, slight movements and not fully alert); awake (alert, ocular opening, minimal motor activity reactive to visual and sound stimulus) and fully awake (eyes open, increased motor activity, maximum reaction to external stimuli and crying stage).

The sleep diary was used just to register the time of feeding and handling by the medical and nursing staff.

### Statistical analysis

The sample size calculation was performed in the WinPEPI program (Programs for Epidemiologists for Windows) version 11.43 and based on the study by Collins et al.^1^. To reach a significance level of 5%, power of 90% and an effect size of at least one standard deviation between the two evaluations, a minimum of 17 premature infants were necessary. Quantitative variables were described by mean and standard deviation or interquartile range and median. Qualitative variables were described by absolute and relative absolute frequencies. To compare means between moments, the t-student test for paired samples was applied. To evaluate the associations of clinical variables with the results of the actigraphy analysis, the Spearman and Mann-Whitney correlation tests were used. SPSS version 21.0 was used to analyze data.

## RESULTS

Table 2 compares the actigraphy findings with and without CPAP. TST, TSP and SE were significantly higher without CPAP. A significant alteration in the sleep macrostructure, with reduction of all the parameters studied (total sleep time, total sleep period and sleep efficiency) was observed, when data were compared to the non-CPAP period.

**Table 2:** Macroarchitecture of sleep with CPAP and without CPAP.

Variables #	Without CPAP	With CPAP	Difference (IC 95%)	p
	Mean ± SD	Mean ± SD		
TST (min)	745,8 ± 125,7	431,8 ± 132,6	313,9 (229,8 to 398,1)	<0,001
TSP (min)	981,9 ± 170,3	691,2 ± 186,6	290,7 (174,5 to 406,9)	<0,001
SE (%)	76,2 ± 6,8	62,0 ± 13,2	14,2 (8,8 to 19,6)	<0,001

Described by mean ± SD; t-student test; TST = Total sleep time; TSP = Total sleep period; SE = Sleep efficiency; SD = Standard deviation; min = minutes.

Table 3 shows the NBAS analysis. The periods in deep and light sleep were significantly higher without nasal CPAP. Drowsiness and waking periods were significantly higher with CPAP.

**Table 3:** Results of the Brazelton - Neonatal Behavioral Assessment Scale.

Variables	With CPAP	Without CPAP	p
	Median (P25-P75)	Median (P25-P75)	
Deep sleep (%)	0 (0-12,8)	25 (7,5-39,8)	0,008
Light sleep (%)	24 (19,8-28,5)	42,5 (31,5-62,5)	0,002
Sleepy (%)	39,5 (29,5-42,8)	12,5 (0-30)	0,001
Awake (%)	29 (12,8-37,5)	0 (0-15)	0,006
Crying (%)	0 (0-1,3)	0 (0-0)	0,206
Fully awake (%)	0 (0-10)	0 (0-0)	0,173

% percentage of time the newborn remained in a certain stage of sleep; Mann-Whitney test.

**Figure 1:**
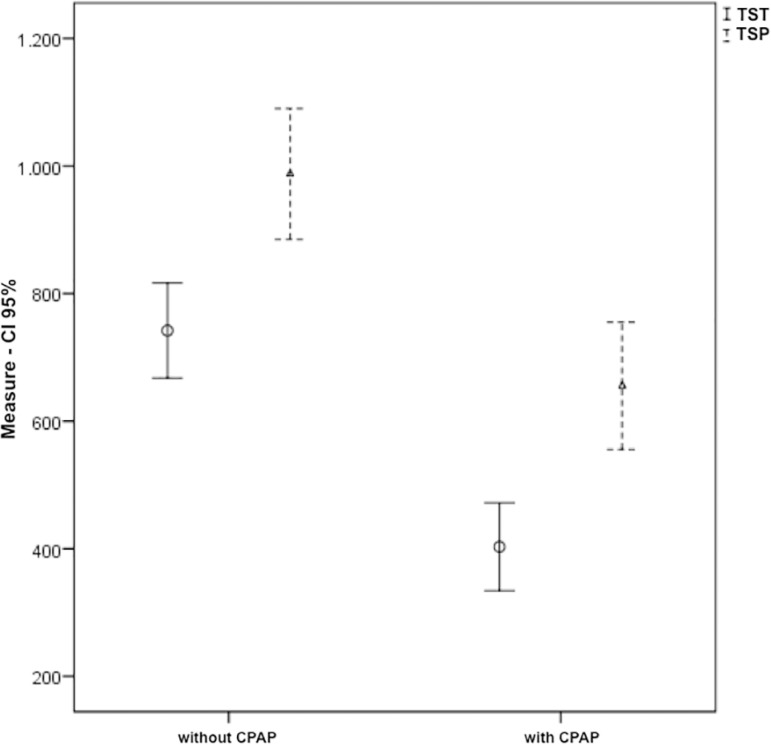
Total sleep time and total sleep period with and without CPAP. Measured in minutes: TST = Total sleep time; TSP = Total sleep period.

**Figure 2:**
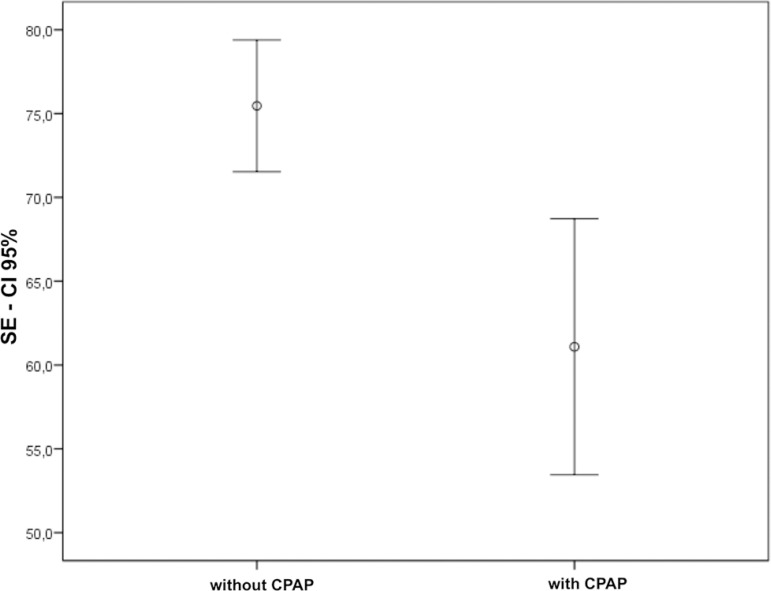
Sleep efficiency with CPAP and without CPAP. SE = Sleep efficiency, measure %.

Results showed a reduction in total sleep time, total sleep period (Figure 1) and sleep efficiency (Figure 2) during the CPAP period when compared to the non-CPAP.

## DISCUSSION

In this study, we have evaluated sleep characteristics of preterm infants through the analysis of two methods, a behavioral scale and the actigraph monitoring. Results showed a reduction in sleep efficiency, total sleep time and total sleep period during the CPAP period when compared to the non-CPAP. NBAS demonstrated significantly greater time of deep sleep and light sleep in the period without CPAP. Both methods identified that CPAP influences preterm sleep expressed as transient alterations in the sleep macrostructure, that were reverted after the withdrawal of the ventilatory support.

The use of nasal CPAP might alter sleep organization in different ways such as due to mechanical problems (incorrect choice of the size of the nasal cannula, problems with its fixation, both essential to provide the necessary pressure in the airways and to avoid lesions in the nasal septum) or lack of constant overview of the device (checking the heating, humidification and flow of oxygen and compressed air)^9^. Further aspects refer to careful changes of body position and the possibility of abdominal distension due to nasal flow and harming ventilator stabilization^9^. Sleep position might also alter sleep organization. In previous studies, Jarus et al.^4^ observed that in the prone position more sleep patterns (deep sleep, light sleep and drowsy) were observed as opposed to more awake patterns (quiet awake, active awake and agitated) that were seen in the supine position. It is important to note that in the NICU where the data was collected there are protocols/routines, regarding care with sounds, lights, handling and position of the newborn that are followed by the assistant team and regularly monitored. It is important to note that, during the 2 hours of infant sleep assessment, staff handling and feeding periods were excluded.

The American Academy of Pediatrics advises that noise levels in the NICU should be less than 45dB. However, it is known that nasal CPAP generates noises greater than 45dB. These values may vary with the type of device and respirators used to deliver continuous positive airway pressure^17,18^.

Reinforcing the findings of our study, previous studies reiterate that any type of manipulation in the baby, lighting and sound can alter the quality of sleep in NICU^4,5,19^. We know that the use of CPAP may increase the manipulation due to an improperly coupled nasal cannula, noises or any physical discomfort suffered by prematurity. Levy et al.^19^ suggests that reducing handling or adjusting the time of handling in active sleep may be beneficial.

Likewise, Collins et al.^1^ compared sleep quality, through actigraphy and behavioral scale, in high-flow catheter and nasal CPAP. This paper observed decreased sleep efficiency and increased activity in the high-flow catheter due to respirator noise and manipulation of the assisting team.

It is important to emphasize that premature infants under CPAP are also under unfavorable clinical conditions, requiring oxygenation and pressure in the upper airway^11,20^. Therefore, the question arises whether CPAP alone is responsible for the alterations on sleep organization or it is an additional factor with the underlying pathology. As we observed in our findings, CPAP withdrawal coincided with a significant improvement in sleep characteristics, but it is important to question whether such improvement is not secondary to a favorable clinical outcome. Further studies are needed to answer this specific question.

As limitations of our study, we can cite the small number of patients despite the power of 90%; another limitation of the study was that the treatment - non-treatment group was not randomized. The sound and handling characteristics of the attending staff should also be considered, since alarms are common at a NICU. In addition, although the NICU had positional routines when using CPAP (alternate prone and supine position), this aspect was not analyzed in this study. No information on circadian rhythmicity were provided or analyzed because actigraph does not show this data.

In conclusion, our study showed that premature infants exposed to nasal CPAP had alterations in the sleep architecture when objective and subjective measures were analyzed.
